# Quantifying the Intra-Regional Precipitation Variability in Northwestern China over the Past 1,400 Years

**DOI:** 10.1371/journal.pone.0131693

**Published:** 2015-07-08

**Authors:** Harry F. Lee, Qing Pei, David D. Zhang, Kan P. K. Choi

**Affiliations:** 1 Department of Geography, The University of Hong Kong, Pokfulam Road, Hong Kong SAR, China; 2 International Centre of China Development Studies, The University of Hong Kong, Pokfulam Road, Hong Kong SAR, China; University of California San Diego, UNITED STATES

## Abstract

There has been a surge of paleo-climatic/environmental studies of Northwestern China (NW China), a region characterized by a diverse assortment of hydro-climatic systems. Their common approach, however, focuses on “deducing regional resemblance” rather than “exploring regional variance.” To date, efforts to produce a quantitative assessment of long-term intra-regional precipitation variability (IRPV) in NW China has been inadequate. In the present study, we base on historical flood/drought records to compile a decadal IRPV index for NW China spanned AD580–1979 and to find its major determinants via wavelet analysis. Results show that our IRPV index captures the footprints of internal hydro-climatic disparity in NW China. In addition, we find distinct ~120–200 year periodicities in the IRPV index over the Little Ice Age, which are attributable to the change of hydro-climatic influence of ocean-atmospheric modes during the period. Also, we offer statistical evidence of El Niño Southern Oscillation (Indo-Pacific warm pool sea surface temperature and China-wide land surface temperature) as the prominent multi-decadal to centennial (centennial to multi-centennial) determinant of the IRPV in NW China. The present study contributes to the quantitative validation of the long-term IRPV in NW China and its driving forces, covering the periods with and without instrumental records. It may help to comprehend the complex hydro-climatic regimes in the region.

## Introduction

Northwestern China (NW China) includes Sha’anxi, Gansu, Ningxia, Qinghai, and Xinjiang, which comprises nearly one-third of China’s territory. The region is typified by aridity; the mean annual rainfall is < 250 mm and annual evaporation is > 1,400 mm [1, 2]. Moreover, the rainy season is short and 50–67% of the yearly total precipitation falls in summer [3]. Scientific research on the subject of the long-term precipitation dynamics in NW China has received considerable attention over the last two decades, as the knowledge of such dynamics is vital for forecasting the societal impacts of precipitation change in the coming decades [4]. However, instrumental precipitation records of NW China only start in AD1951 and are too brief to investigate multi-decadal or longer hydro-climatic oscillations and to evaluate the magnitude and frequency of contemporary droughts in a long-term context [5]. Fortunately, there has been a surge of fine-grained regional moisture/precipitation reconstructions for NW China (in annual to decadal resolution) in recent years, which are mainly derived from proxies such as tree-ring chronologies [4, 6–15], cave speleothems [16–18], ice cores [19, 20], and lake sediments [21, 22]. They help to unveil the complex climate dynamics in different parts of NW China over extended periods.

**Fig 1:**
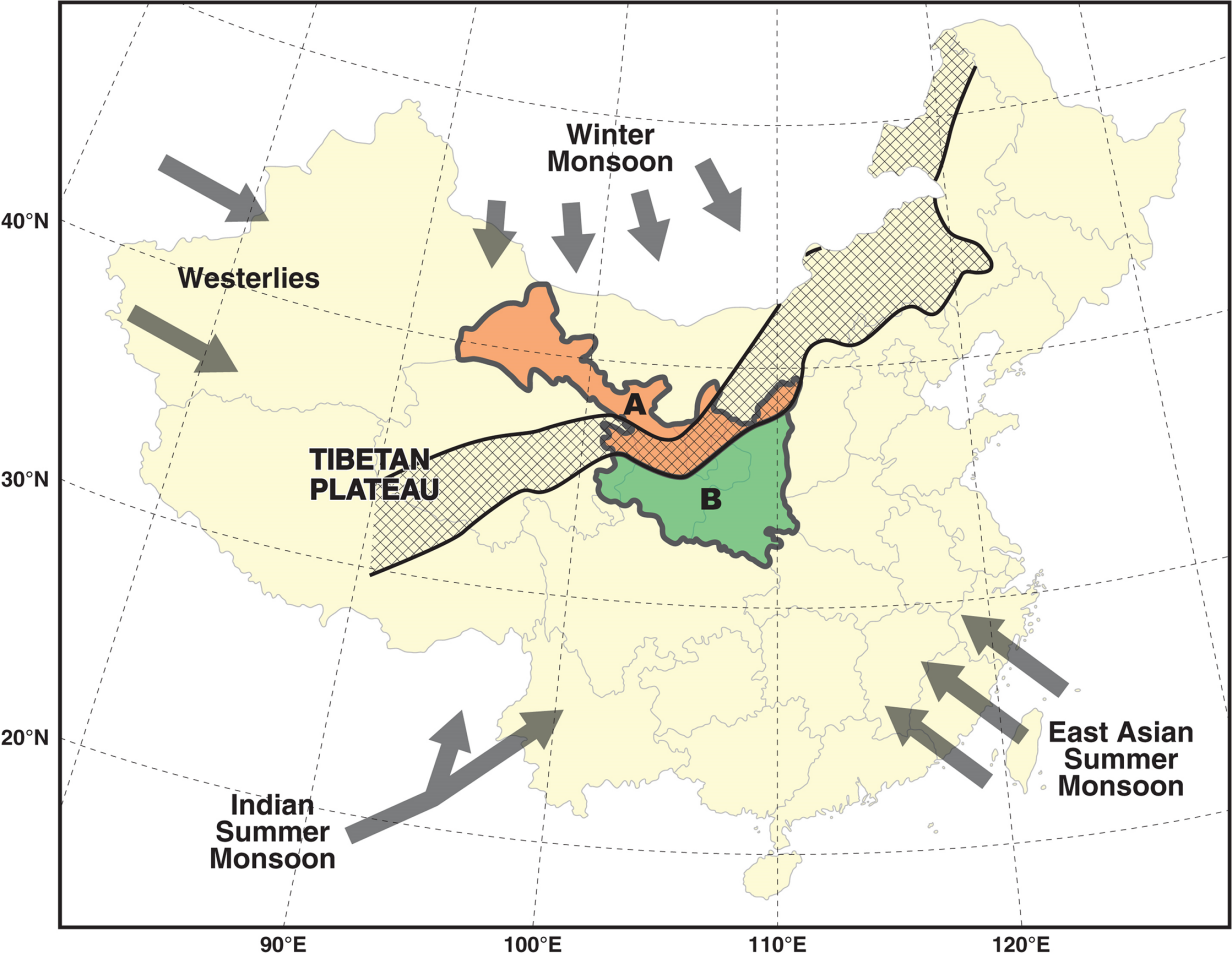
Location and geographic configuration of our study area (modified from [32–34]). Our study area is delineated into two regions (Regions A and B) according to the present-day 400 mm isohyet. The hydro-climate in Regions A and B is dominated by the Westerlies and ASM, respectively. Arrows represent ASM (including East ASM and Indian Summer Monsoon), Westerlies, and Winter Monsoon. The checkered belt is the region between the 200–400 mm isohyets, which is the approximate present-day northern fringe of ASM.

Nevertheless, the availability of proxy data depends on the properties of the proxy media itself. For instance, trees for dendrochronology tend to concentrate in places favorable to plant growth. Also, the distribution of cave speleothems, ice cores, and lake sediments are confined to certain geographic regions. For the selection of study areas, those sites in which the proxies contain very strong climate signals are usually preferred. The resulting reconstructions may be ideal to reveal the condition at climate-sensitive/marginal areas. Yet, they may contain site-specific climatic signals [23]. It remains an issue how far the associated findings can be generalized to the other parts of NW China. It is worth mentioning that NW China sits at the present-day northern fringe of the Asian Summer Monsoon (ASM) (Fig 1). The hydrological balance and effective moisture of the region is controlled by the interactions of ASM, Winter Monsoon, and Westerlies [2, 24]. Subject to this unique geographic configuration, precipitation regimes in NW China are typified by salient intra-regional differences, especially between the zones north and south of the ASM fringe [25–27]. This inherent feature makes it impossible to apply generalizations about the precipitation regime of any single locality to other parts of NW China. Most importantly, a fine-grained picture of the intra-regional precipitation variability (IRPV) in NW China over extended periods is still nonexistent at the moment, as the common approach employed in most of the abovementioned paleo-climatic/environmental studies has focused on “deducing regional resemblance” rather than “exploring regional variance.” This limits the examination of the related phenomena.

Historical documents are another proxy media for high-resolution paleo-climate reconstruction. Chinese historical documents are accurate in chronology and explicit in their description of dry–wet and warm–cold conditions [23]. They are useful in locating disaster events in a long time span, particularly when the events are recorded on an annual basis [28]. Also, they contain a wide spectrum of climate variability and encompass the evidence of past changes at a range of time-scales. In previous studies, Yan et al. [29] rely on historical documents to compile the drought/flood index of the Hai River and Xi’an region over the last two millennia. Tan et al. [24] extract climate information from local historical documents in Longxi (which consists of Lanzhou area, Dingxi area, Wushan County, Huining County, Gangu County, and Qin’an County in the present days), the northeastern margin of the Tibetan Plateau, and provide a millennium span of precipitation variability. Recently, the authors employed the dryness/wetness grade series of 19 sites in NW China, which are primarily derived from historical documents, to reconstruct the annual geographic extent of drought anomalies in NW China in AD1470–2008 [30]. Given the potential of historical records in the studies of climate history in NW China, in this study, we base our research on historical documents to quantify the IRPV in NW China over an extended period and find the major driving forces behind it, which is the first-ever attempt to do so.

## Materials and Methods

### Historical hydro-climatic records, study area, and study period

We focus on Yuan’s [31] detailed inventory of flood/drought disasters, *Disaster History of Northwestern China*, spanning AD580–1979, to trace the IRPV in NW China. Yuan [31] relies on a careful survey of 685 volumes of various official dynastic histories, local chronicles, and instrumental data from local meteorological stations to tabulate flood/drought disasters for Sha’anxi, Gansu, Ningxia, and eastern Qinghai. His dataset also contains information about the areas affected by those disasters in present-day place names and is believed to be one of the most comprehensive historical hydro-climatic records for NW China. The inventory has been applied in our previous studies see [32, 33, 34]. To further ensure the accuracy for the hydro-climatic records employed in this study, those records in earlier periods in Yuan’s [31] dataset are cross-checked with their original sources as far as possible. The records in later periods are compared with the *Yearly Charts of Dryness/Wetness in NW China for the Last 500-year Period* (1470–2008) compiled by Bai et al. [35]. The work of Bai et al. [35] is the official updated version of widely-employed *Yearly Charts of Dryness/Wetness in China for the Last 500-year Period* [36]. The latter dataset contains dryness/wetness grade series for 120 sites in China in AD1470–1979, and 12 of the sites are located in NW China (including Xining, Geermu, Zhangye, Lanzhou, Pingliang, Tianshui, Yinchuan, Yulin, Yan’an, Xi’an, Hanzhong, and Ankang). In recent years, Bai et al. [35] have updated the dryness/wetness grade series of those 12 sites, and have added the dryness/wetness grade series of seven new sites in NW China into the dataset (including Xinghai, Yushu, Gangcha, Wudu, Yanchi, Guyuan, and Baoji). Our principle is that those flood/drought records in Yuan’s [31] inventory will be dropped if they do not match with their original sources or Bai et al.’s [35] yearly charts.

Subject to the geographic coverage of historical flood/drought records in Yuan’s [31] inventory, our study area is confined to Sha’anxi, Gansu, Ningxia, and eastern Qinghai (Fig 1). Our study area is believed to be a reasonable representation of NW China because it covers > 50% of the inhabited area in NW China. Our study time span is delimited to AD580–1979, which is identical to the time span of Yuan’s [31] inventory.

### Quantification of the IRPV in NW China

We take the following steps to quantify the IRPV in NW China:

First, we divide our study area into two macro regions according to the approximate present-day 400 mm isohyet, which is the threshold value in distinguishing the arid/semi-arid region from the humid region in NW China. The 400 mm isohyet is also the natural divide between animal husbandry and agricultural regions [37]. The region delineation is shown graphically in Fig 1. The annual precipitation in Region A is < 400mm, while that in Region B is > 400mm. The associated geographic units of the two regions are detailed in Tables 1 and 2, respectively.

**Table 1:** Geographic units in Regions A and B.

Region A	Region B
Yulin Region, Sha’anbei (Sha’anxi)	Yan’an Region, Sha’anbei (Sha’anxi)
Dingxi Region, Longzhong (Gansu)	Baoji City, Guanzhong (Sha’anxi)
Lanzhou City, Longzhong (Gansu)	Xianyang City, Guanzhong (Sha’anxi)
Linxia Region, Longzhong (Gansu)	Xi’an City, Guanzhong (Sha’anxi)
Wuwei Region, Hexi (Gansu)	Tongchuan City, Guanzhong (Sha’anxi)
Zhangye Region, Hexi (Gansu)	Weinan Region, Guanzhong (Sha’anxi)
Jiuquan Region, Hexi (Gansu)	Hanzhong Region, Sha’annan (Sha’anxi)
Yinchuan City, Ningbei (Ningxia)	Ankang Region, Sha’annan (Sha’anxi)
Shizuishan City, Ningbei (Ningxia)	Shangluo Region, Sha’annan (Sha’anxi)
Yinnan Region, Ningbei (Ningxia)	Qingyang Region, Longdong (Gansu)
Guyuan Region (Ningxia)	Pingliang Region, Longdong (Gansu)
Haidong Region, Haidong (Qinghai)	Tianshui City, Longnan (Gansu)
	Longnan Region, Longnan (Gansu)
	Gannan Region, Longzhong (Gansu)
	Huangnan Region, Haidong (Qinghai)

**Table 2:** Decades with extremely high/low IRPV index values.

Climatic episode	Extremely high IRPV	Extremely low IRPV
Pre-MWP, AD580–1000	AD740–749	AD810–819
		AD980–989
MWP, AD1000–1300	AD1110–1119	AD1010–1019
	AD1260–1269	AD1150–1159
	AD1270–1279	AD1230–1239
		AD1280–1289
MWP–LIA Transition,	AD1300–1309	AD1390–1399
AD1300–1400	AD1350–1359	
LIA, AD1400–1900	AD1440–1449	AD1410–1419
	AD1560–1569	AD1470–1479
	AD1580–1589	AD1510–1519
	AD1590–1599	AD1640–1649
	AD1600–1609	AD1660–1669
	AD1690–1699	AD1670–1679
	AD1730–1739	AD1680–1689
	AD1790–1799	AD1700–1709
	AD1800–1809	AD1810–1819
		AD1830–1839
Warm 20^th^ century,	—-	—-
AD1900–1979		

Second, in reference to previous studies [24, 38], we base on flood/drought disasters to identify flood/drought years in the two regions. Then, we apply the following formula to convert those flood/drought years into precipitation indices for Region A (PI_A_) and Region B (PI_B_):

PI=2(F−D)/(F+D) 1

where F and D represent the decadal frequencies of flood and drought years, respectively. The index is in decadal units. If the number of flood and drought years is equal, then the PI is equal to zero (also if no flood and drought events are measured); if the climate is wet (dry), the PI is greater (smaller) than one. Usually, the resulting index values will be further converted to five grades according to some threshold values. However, this practice may sacrifice some signals embedded in time-series. Therefore, we skip this step when composing the precipitation indices.

Third, in reference to the approach employed by Osborn and Briffa [39] in determining the geographic extent of climatic anomalies, we employ the following formula to compose an index quantifying the IRPV in NW China:

$$IRPV=PI_{A}-PI_{B}$$ 2

**Fig 2:**
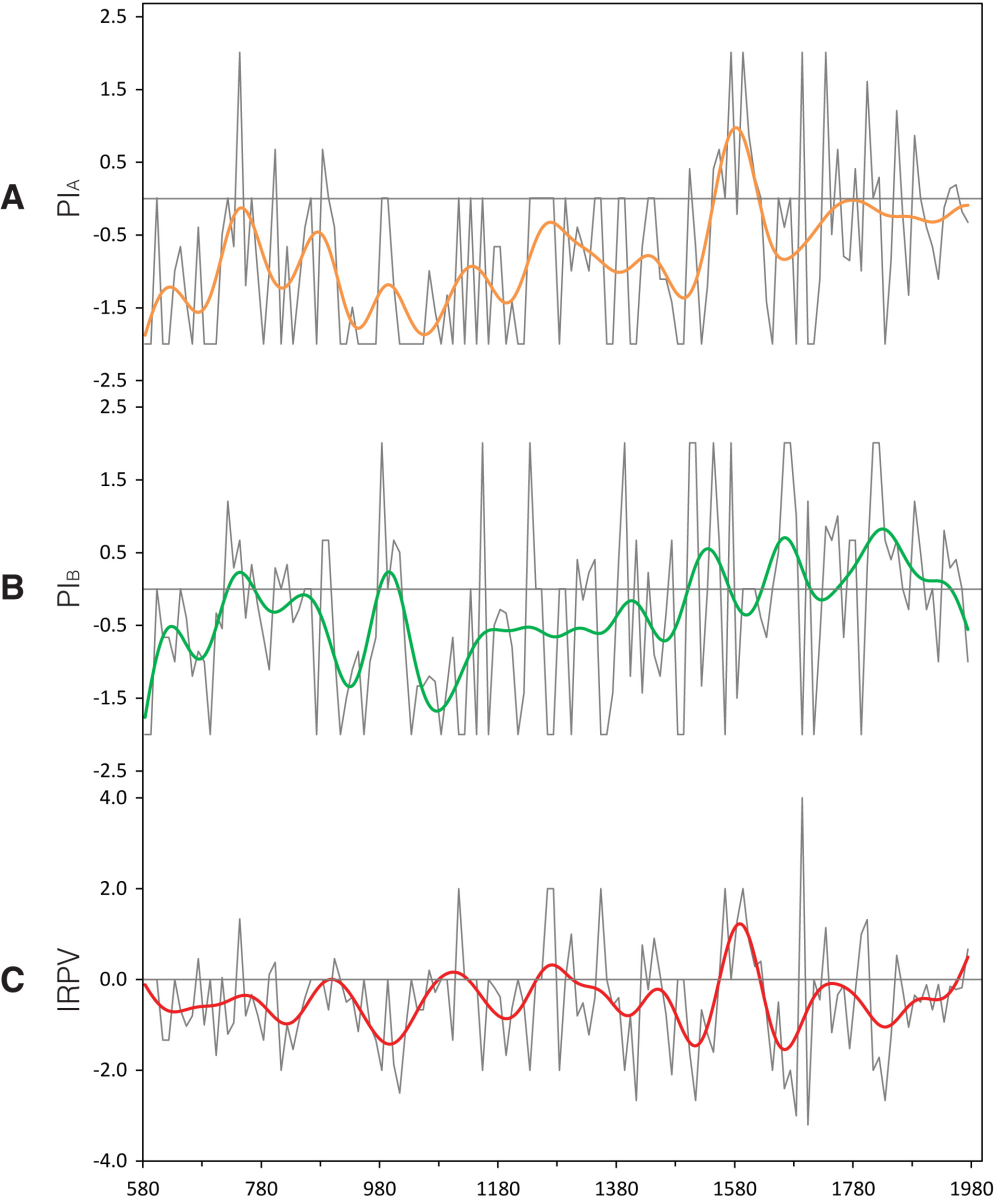
PI_A_, PI_B_, and the IRPV index. In this study, we base on historical flood/drought records to reconstruct (**A**) PI_**A**_ and (**B**) PI_**B**_. We subtract PI_**B**_ from PI_**A**_ to give (**C**) the IRPV index. All of the above indices are in decadal units and spanned AD580–1979 (see Section 2.1). All of the time-series are smoothed by Butterworth 10-pt (1-pt = 10 year) low-pass adaptive filter.

The index, which is shown in Fig 2C, represents the relative change of precipitation between Regions A and B, with the direction and the magnitude of precipitation change taken into account. If the precipitation change in the two macro regions moves in opposite directions and becomes more extreme, the index value will be positively/negatively deviated away from zero, and *vice versa*. A positive index value means that Region A is getting more humid in comparison to Region B, and *vice versa*. The complete PI_A_, PI_B_, and IRPV index are available in Supporting Information S1 Table.

### Data about the driving forces of IRPV

Recent studies also demonstrate the effect of various ocean-atmospheric modes and their related factors in driving pluvial events in different localities of NW China [6–9, 11, 18, 24, 32, 33, 40]. In this study, we seek to identify which factors are primarily responsible for the long-term IRPV in NW China via statistical means. Our data of various factors are retrieved from the webpages of NOAA’s National Climatic Data Centre (http://www.ncdc.noaa.gov/data-access/paleoclimatology-data/datasets/climate-reconstruction) and also KNMI Climate Explorer (http://climexp.knmi.nl). As our IRPV index is in decadal resolution (cf. Quantification of the IRPV in NW China), those data will be transformed into decadal units prior to statistical analysis.

### Wavelet analysis

For climatic systems, transient dynamics appear to be the rule rather than the exception in nature, because climatic processes are influenced by exogenous trends or pre-dominated by complex endogenous dynamics. Hence, it may be inappropriate to apply traditional techniques (which make the assumption that the statistical properties of the time-series do not vary with time) to analyze non-stationary climatic variables or their mutual dependencies. Wavelet analysis is a powerful tool that is already in use throughout science and engineering. By performing a local time-scale decomposition of the signal in time-series, wavelet analysis is germane in analyzing non-stationary systems [41–43]. The continuous wavelet transform decomposes the time-series into both time and frequency components, the calculation of the wavelet power spectrum quantifies in the time-frequency domain the distribution of the variance of the time-series [43, 44].

There are two major methods of wavelet analysis, namely wavelet spectral analysis and wavelet coherency analysis. In this study, we employ wavelet spectral analysis to compose the continuous wavelet power spectrum of time-series in order to track how its periodic components of signal change over time [43, 45]. At the same time, we employ wavelet coherency analysis to present coherencies between two time-series, in order to identify any significant associations between two time-series of specific frequency-time domain [43, 44]. For the above methods, Morlet wavelet is employed to decompose signals, which is generally regarded as an efficient means of detecting variations in the periodicities of geophysical signals along time-series in a continuous manner [46].

### Delineation of major climatic episodes

There is not any universal consensus about the exact time spans of the Medieval Warm Period (MWP) and the Little Ice Age (LIA) in China. To facilitate the comparison between our findings and those in other studies, we employ the delineation offered by the Intergovernmental Panel on Climate Change [47]: The MWP spans AD1000–1300, while the LIA spans AD1400–1900. The above delineation matches with the warm and cold phases in the reconstructions of China-wide temperature [48, 49] and is applied in recent studies as well [27].

## Results

### Validation of IRPV index

#### Data structure

As historical flood/drought records in Yuan’s [31] inventory are extracted from diverse sources, and the availability of records increases with time, we employ the Augmented Dickey-Fuller (ADF) test to check whether the data structure of our IRPV index, which is derived from Yuan’s [31] inventory, is temporally consistent. The test is expressed in the following equation [50]:

$$DY_{t}=\mu +\delta Y_{t-1}+\beta_{1}DY_{t-1}+\beta_{2}DY_{t-2}+\ldots +\beta_{p}DY_{t-p}+\varepsilon_{t}$$ 3

$$DY_{t}=Y_{t}-Y_{t-1}$$ 4

Where *μ* is a constant, *δ* is a unit root, *β* is the coefficient on a time trend, and *p* is the lag order of the autoregressive process. Our null hypothesis is that the data structure of IRPV time-series is non-stationary. The ADF test is operationalized via the statistical software EViews, with three possible conditions of data structure considered: no intercept and no trend, with intercept but no trend, with both intercept and trend. Our test results show that the null hypothesis can be rejected at the 0.01 significance level in all of the above conditions, indicating that the data structure of our IRPV time-series is temporally consistent.

#### Comparison with other paleo-climatic records

**Fig 3:**
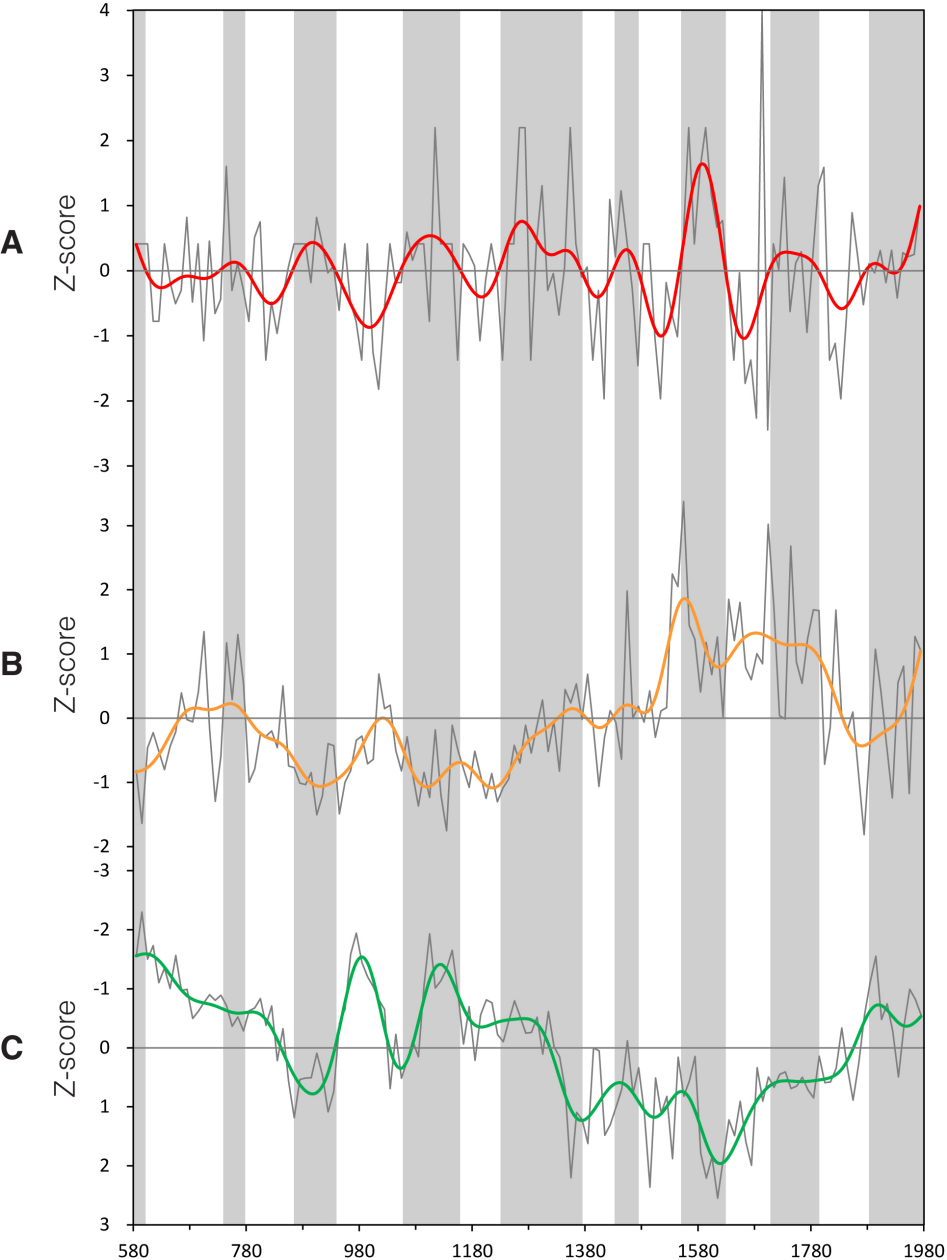
Comparison of the IRPV index with other paleo-climatic records, AD580–1979. (**A**) IRPV index. (**B**) Ice accumulation in Guliya ice cap on Tibetan Plateau [19]. (**C**) ASM precipitation index derived from Wanxiang Cave, Southern Gansu [17]. All of the above indices are in normalized decadal units and smoothed by Butterworth 10-point (decade) low-pass filter. The y-axis of (**C**) is inverted as its original measurement unit is negatively correlated with the amount of precipitation. Grey shades represent the periods in which Region A is becoming more humid in comparison to Region B as indicated in the IRPV index.

**Fig 4:**
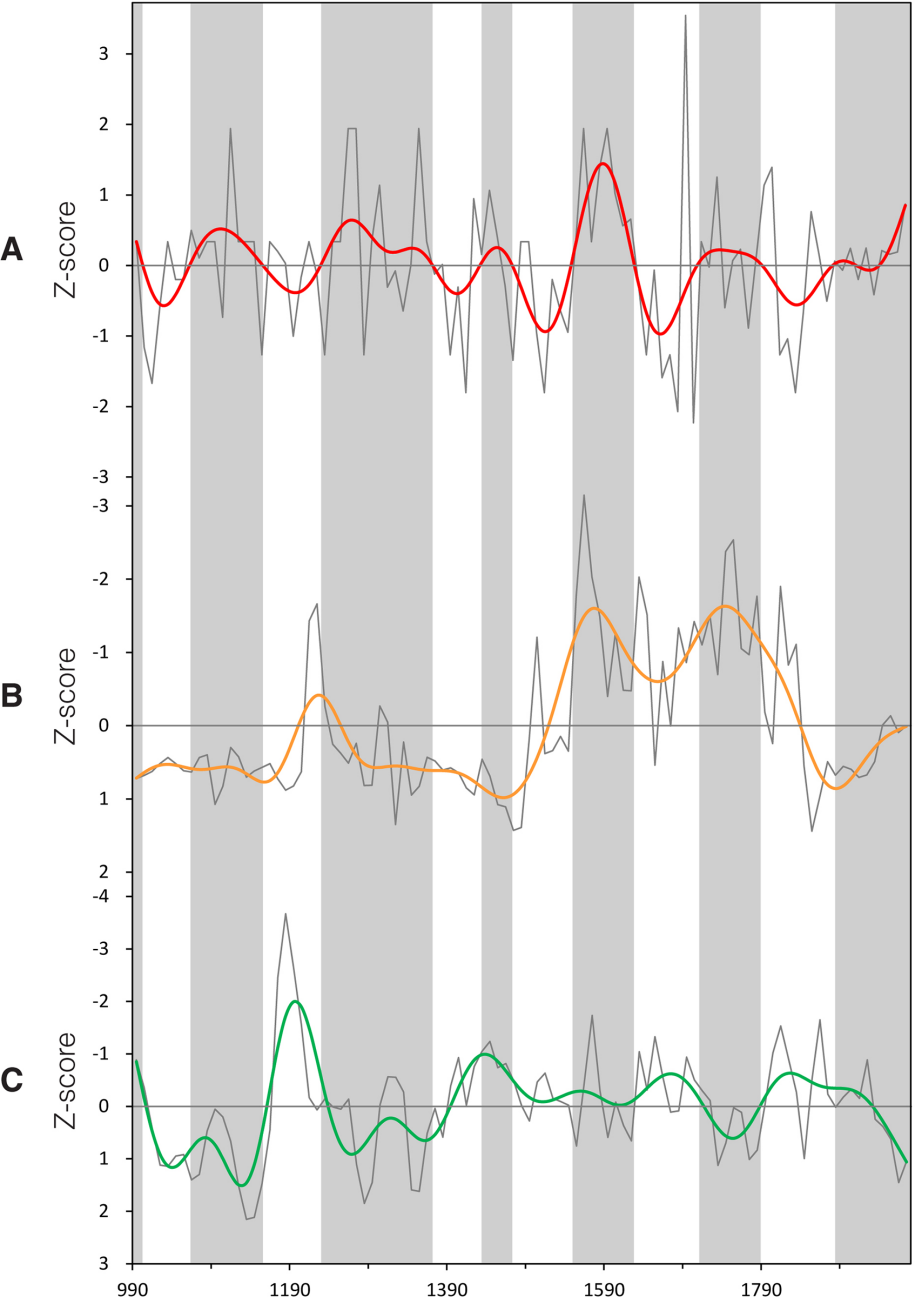
Comparison of the IRPV index with other paleo-climatic records, AD990–1979. (**A**) IRPV index. (**B**) Chironomid-based salinity reconstruction from varved sediments in Sugan Lake, Qaidam Basin [21]. (**C**) ASM precipitation index derived from Jiuxian Cave, Sha’anxi [18]. All of the above indices are in normalized decadal units and smoothed by Butterworth 10-point (decade) low-pass filter. The y-axes of (**B**) and (**C**) are inverted as their original measurement units are negatively correlated with the amount of precipitation. Grey shades represent the periods in which Region A is becoming more humid in comparison to Region B as indicated in the IRPV index.

**Fig 5:**
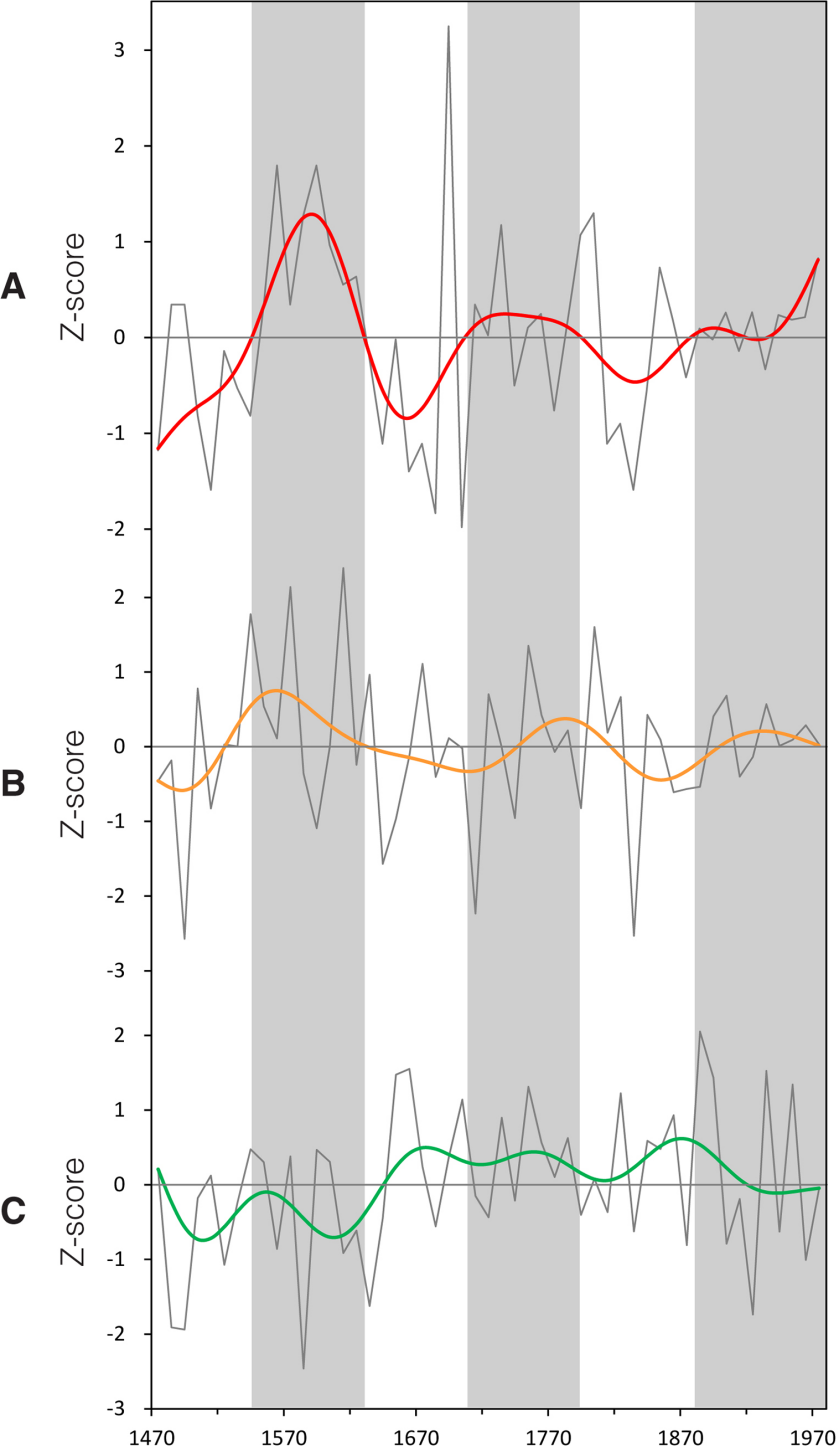
Comparison of the IRPV index with other paleo-climatic records, AD1470–1979. (**A**) IRPV index. (**B**) Palmer Drought Severity Index in Region A derived from the *Monsoon Asia Drought Atlas* [51]. (**C**) Precipitation index of North-central China [52]. All of the above indices are in normalized decadal units and smoothed by Butterworth 10-point (decade) low-pass filter. Grey shades represent the periods in which Region A is becoming more humid in comparison to Region B as indicated in the IRPV index.

To further verify the validity of our IRPV index, we compare it with three different pairs of paleo-climatic records that are derived from various proxy media in opposite locations in NW China (i.e., Westerlies-dominated region versus ASM-dominated region). The pair up is made according to the geographic domains (i.e., spatial coverage and location) and data time-span of those records. The first pair includes ice accumulation record in the Guliya ice cap on the Tibetan Plateau [19] and ASM precipitation reconstruction derived from the speleothem in southern Gansu [17]. The two indices cover our entire study time-span (i.e., AD580–1979) (Fig 3). The second pair consists of chironomid-based salinity reconstruction derived from varved sediments in Qaidam Basin [21] and ASM precipitation index derived from the speleothem in Sha’anxi [18]. Their values over their common period (i.e., AD990–1979) are put against our IRPV index for comparison (Fig 4). For the third pair, we average the Palmer Drought Severity Index of the grids (in 2.5° x 2.5° resolution) over Region A in *Monsoon Asia Drought Atlas* that is derived from a network of Asian tree-ring data [51]. We then pair it with precipitation reconstruction over North-central China (in Region B) that is derived from historical documents and tree-ring data [52] (Fig 5). The third pair of paleo-climatic records is relatively short (AD1470–1979), but corresponded well to the IRPV index in terms of their geographic domains. Prior to the comparison, all of the abovementioned series are transformed into decadal resolution, normalized in pairs into common units over the period concerned, and with their centennial variability elicited by Butterworth 10-point (decade) low-pass filter. Our result shows that at the centennial time-scale, when Region A is getting more humid in comparison to Region B as indicated in our IRPV index, the same feature can be envisaged in the three pairs of paleo-climatic records in general (Figs 3–5). Briefly, our IRPV index captures the footprints of intra-regional precipitation variation in NW China. Such variation is also valid over larger geographic areas at longer time-scales [25–27].

#### Extreme values

We apply a bootstrapping procedure to identify those decades that have extremely high/low IRPV index values. Our IRPV index time-series is randomly sampled (with replacement) to generate 10,000 new time-series, with *n* equal to the total number of data points of our original time-series. Then, the 90% and 10% quantiles of the confidence limit of every bootstrapped time-series are calculated. Finally, we average all of the generated 90% and 10% quantiles to obtain the final confidence limit for our IRPV index. The above method allows us to estimate empirically the sampling distribution of statistic without making assumptions about the form of the population and without deriving the sampling distribution explicitly [53]. We base on the bootstrapped confidence limit to screen out those anomalous decades (i.e., IRPV is > 90% or < 10% quantile of the confidence limit), which are listed in Table 2. It is observed that those decades are clustered in the LIA, implying that the geographic pattern of hydro-climate in NW China was very unstable during the time.

#### Wavelet power spectrum

**Fig 6:**
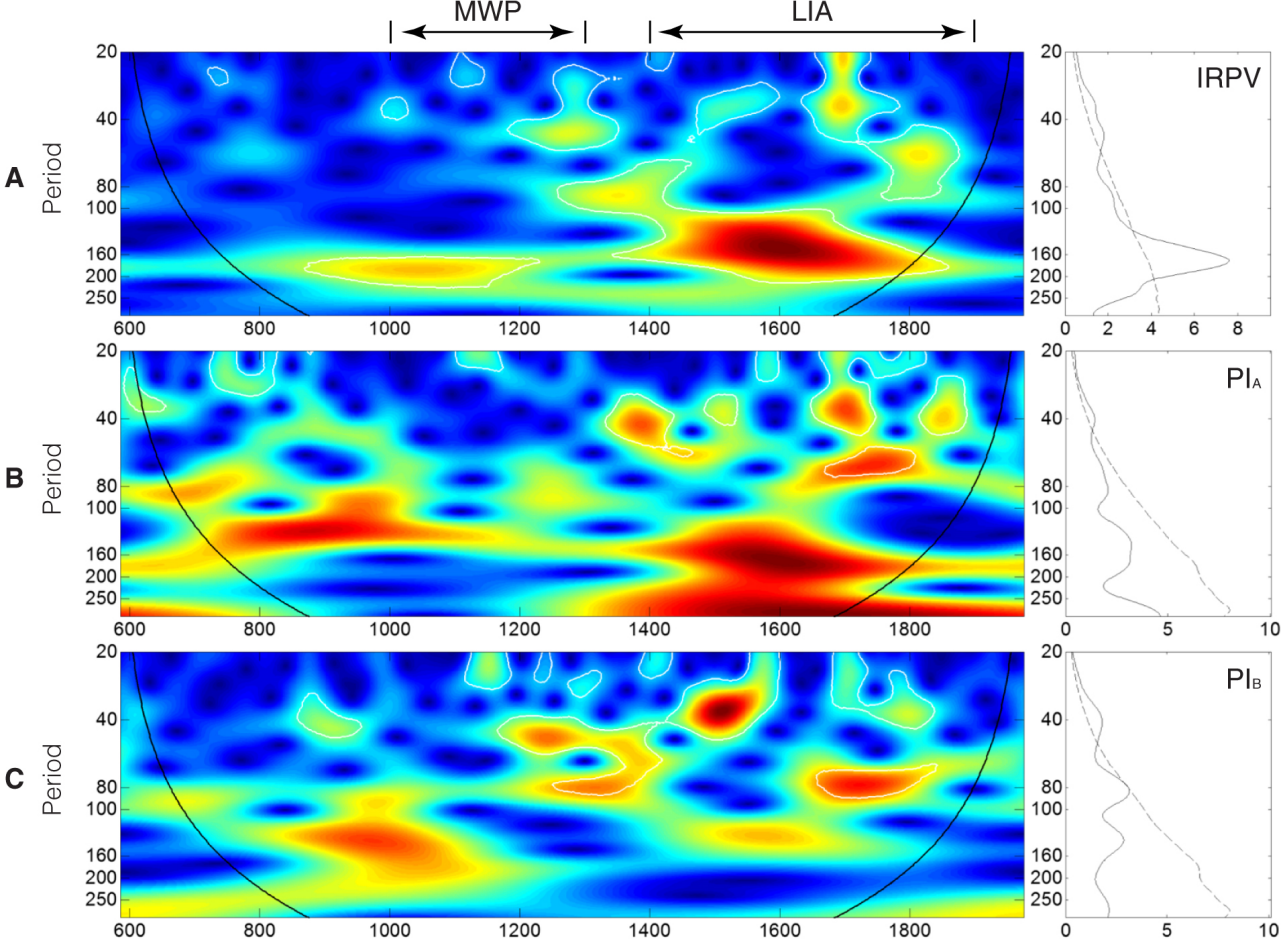
Continuous wavelet power spectra and average wavelet power spectra of the IRPV index, PI_A_, and PI_B_. (**A**) IRPV index (**B**) PI_**A**_. (**C**) PI_**B**_. For the left graphs of **A**–**C**, continuous wavelet power spectra of the reconstructions are shown. The color code for power values varies from dark blue (low values) to dark red (high values) and the black curve indicates the cone of influence that delimits the region not influenced by edge effects. For the right graphs of **A**–**C**, the average wavelet power spectra of the reconstructions are presented. The dashed lines show the α = 5% significance levels computed based on 1,000 Markov bootstrapped series. P-values associated with the values within the region delineated by the dashed line are < 5%.

The resolution of our IRPV index time-series is enhanced by linear interpolation and then wavelet transformed to divulge its main periodicities and the evolution in time of each frequency [54]. The wavelet power spectrum of the IRPV index time-series reveals that there are significant ~20–60 and ~120–200 year bands (Fig 6A). The two year bands enclose the significant periodicities of monsoon rainfall in China [18, 55] and India [56, 57], implying that the IRPV in NW China is closely connected with ASM. As our IRPV index is about the relative precipitation change between the regions delineated by the present-day 400 mm isohyet (Fig 1), which is also the approximate fringe of ASM in NW China, the match of the periodicities may in some way evidence the validity of the index. It is worth mentioning that the ~120–200-year frequency band is particularly strong in the LIA. Its time span is also coincident with the temporal cluster of extremely high/low IRPV index values (see Fig 2C and Table 2). This reveals the hydro-climatic regime in NW China in the LIA to be associated with remarkably strong fluctuation of the IRPV and instability, setting it apart from the rest in the past 1,400 years. We compare the wavelet power spectrum of the IRPV index with those of PI_A_ and PI_B_ (Fig 6B–6C). Although the same ~120–200-year periodicities are also observed in the two regions, it is much more apparent in Region A. Previous studies show that the hydro-climatic records along the eastern-southern fringe of the arid region in NW China are associated with unusual wet–dry oscillation during the LIA [58]. In addition, the hydro-climate in Qaidam Basin, which is adjacent to Region A, is also highly unstable during the period [21]. This indicates that the unusual ~120–200-year periodicities are largely attributable to the hydro-climatic anomalies in Region A.

### Driving forces of IRPV in NW China

#### ASM

**Fig 7:**
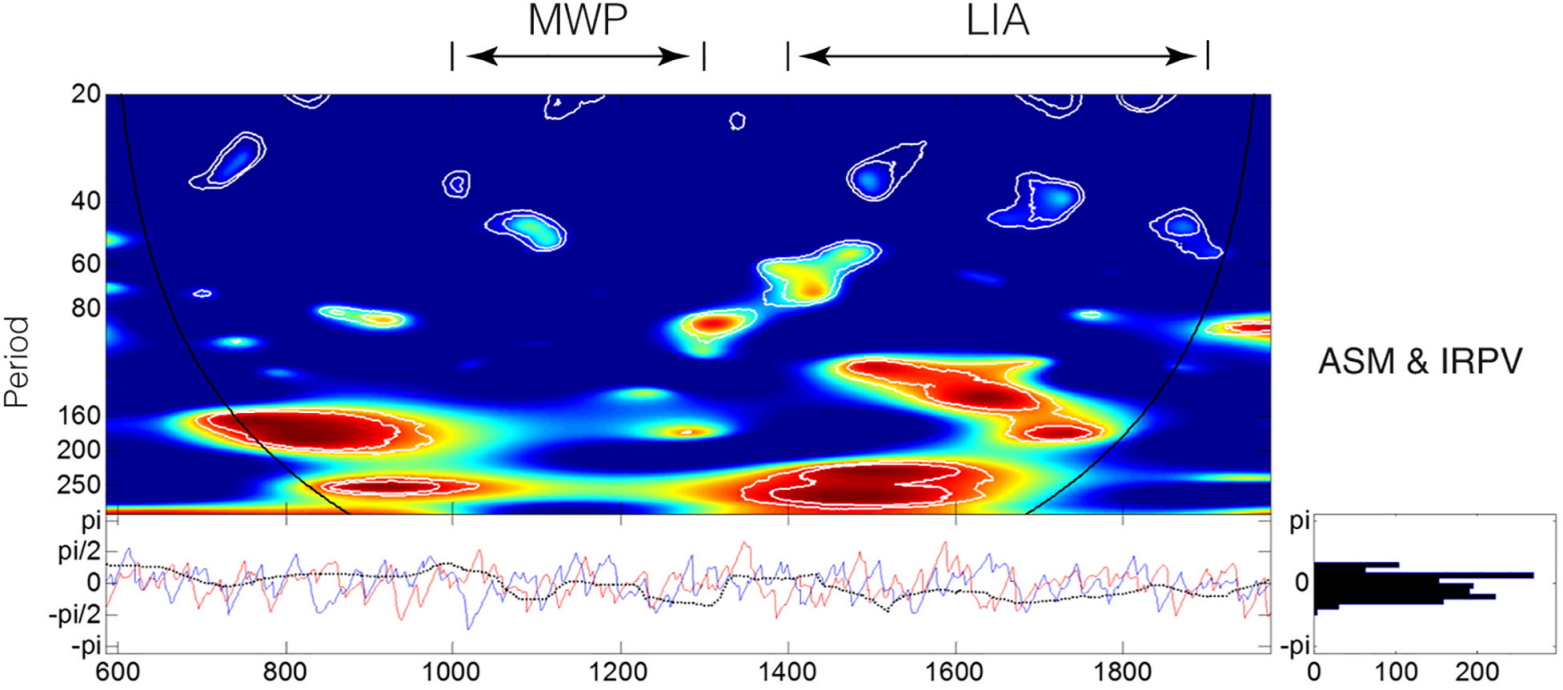
Wavelet coherency between the IRPV index and ASM [17]. For the upper-left graph, the color code for coherence values varies from dark blue (low values) to dark red (high values). The black curve indicates the cone of influence that delimits the region not influenced by edge effects and the dashed line show the α = 10% significance levels computed based on 1,000 Markov bootstrapped series. For the lower-left graph, the dotted lines represent phase difference; the red line represents the phase of ASM; and the blue lines represent the phase of IRPV. For the lower-right graph, the distribution of the phase difference of the two considered time-series is shown.

Our study area covers both the Westerlies-dominated and ASM-dominated regions (i.e., Regions A and B). As the variation of monsoon precipitation is generally regarded one of the most important factors in driving the precipitation disparity between the two regions [25, 59], we compare the IRPV index with the strength of ASM, which is the major determinant of monsoon precipitation. The employed ASM reconstruction is derived from the stalagmite oxygen isotope in Wanxiang Cave in Gansu and spanned AD192–2003 [17]. The result shows that there are significant 160+ year periodicities in the ASM–IRPV coherency (Fig 7). However, the periodicities are intermittent, which are relatively weak in the MWP and the warm 20^th^ century. The above findings indicate that in general, the IRPV in NW China is driven by the fluctuation of monsoon precipitation. Yet, subject to the intermittence of the periodicities in the ASM–IRPV coherency (Fig 7), together with the fact that the monsoon precipitation in NW China is simultaneously modulated by, or interacts with, various ocean-atmospheric modes [6–9, 11, 18, 24, 32, 33, 40], it is necessary to examine whether the variability of IRPV index is also determined by the ocean-atmospheric modes.

#### Ocean-atmospheric modes

**Fig 8:**
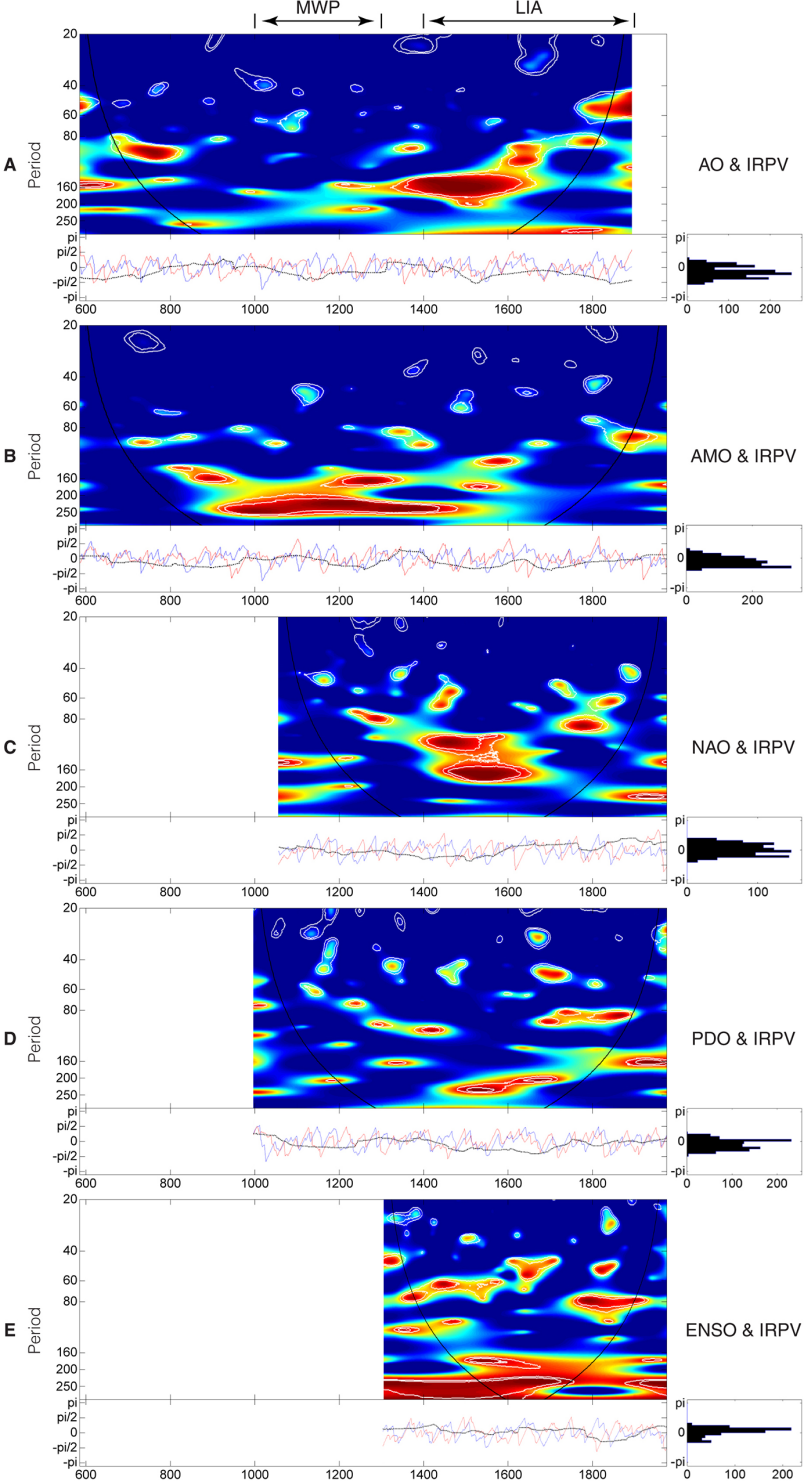
Wavelet coherency between the IRPV index and ocean-atmospheric modes. (**A**) AO [28] and the IRPV. (**B**) AMO [60] and the IRPV. (**C**) NAO [61] and the IRPV. (**D**) PDO [62] and the IRPV. (**E**) ENSO [63] and the IRPV. For the upper-left graphs of **A**–**E**, the color code for coherence values varies from dark blue (low values) to dark red (high values). The black curve indicates the cone of influence that delimits the region not influenced by edge effects and the dashed line show the α = 10% significance levels computed based on 1,000 Markov bootstrapped series. For the lower-left graphs of **A**–**E**, the dotted lines represent phase difference; the red line represents the phase of the ocean-atmospheric mode considered; and the blue lines represent the phase of IRPV. For the lower-right graph of **A**–**E**, the distribution of the phase difference of the two considered time-series is shown.

We go on to measure the coherency between the IRPV index and various ocean-atmospheric modes reconstructions, such as Arctic Oscillation (AO, developed from historical snow anomaly events in Eastern Asia and spanned AD1–1899) [28], Atlantic Multi-decadal Oscillation (AMO, derived from a global climate proxy network and spanned AD500–2006) [60], North Atlantic Oscillation (NAO, defined as the difference of the aridity threshold proxies between Scotland and Morocco that are the opposing poles of NAO, and spanned AD1049–1995) [61], Pacific Decadal Oscillation (PDO, developed from tree-ring chronologies in California and Alberta that are the opposite ends of the PDO precipitation dipole, and spanned AD993–1996) [62], and El Niño Southern Oscillation (ENSO, derived from tree-ring chronologies in Asia, New Zealand, and North and South America and spanned AD1301–2005) [63] (Fig 8A–8E). Two findings are highlighted. First, out of various multi-decadal to centennial frequency bands presented in Fig 8, the ~60–80 year frequency band for the ENSO–IRPV coherency is shown to be relatively more conspicuous and continuous. Only during the Maunder Minimum (c. AD1645–1715) [64], which is also the coldest period over the past millennia [65, 66], are its periodicities shortened to ~50–60 years (Fig 8E). This reveals ENSO to be the prominent driver of IRPV in NW China at the multi-decadal to centennial time-scale. Second, a different hydro-climatic regime in NW China during the LIA is observed. In the MWP and its neighboring periods, there are strong 160+ year periodicities in the AMO–IRPV coherency. But, the periodicities weaken in the LIA (Fig 8B). In parallel, during the LIA, there are distinct ~160–200 year and ~120–200 year periodicities in the AO–IRPV coherency and the NAO–IRPV coherency, respectively (Fig 8A and 8C). The synchronicity of those distinct periodicities indicates the strong AO-NAO coupling [28] as well as strong hydro-climatic influence from the Atlantic and the Arctic Oceans upon NW China during the time. Besides, it also suggests that the hydro-climatic regime of NW China during the LIA is atypical, which may account for the presence of the anomalous ~120–200 year frequency band over the LIA in the IRPV index (Fig 6A). Furthermore, the distinct periodicities in the NAO–IRPV coherency corroborates our previous findings about the out-of-phase hydro-climatic influence of NAO in NW China during the LIA [32, 33].

For the ENSO–IRPV coherency, there are also strong 160+ year periodicities (Fig 8E). But, our employed ENSO record [63] is only ~700 years in length, which may be a bit short for confirming such low-frequency variability. As ENSO is engendered by the coupling variations in the sea surface temperature (SST) of the Eastern Tropical Pacific Ocean and in air surface pressure in the Western Tropical Pacific Ocean [67], we proceed to examine the influence of Equatorial Pacific SST upon the IRPV in NW China.

#### Equatorial Pacific SST and China-wide land surface temperature

**Fig 9:**
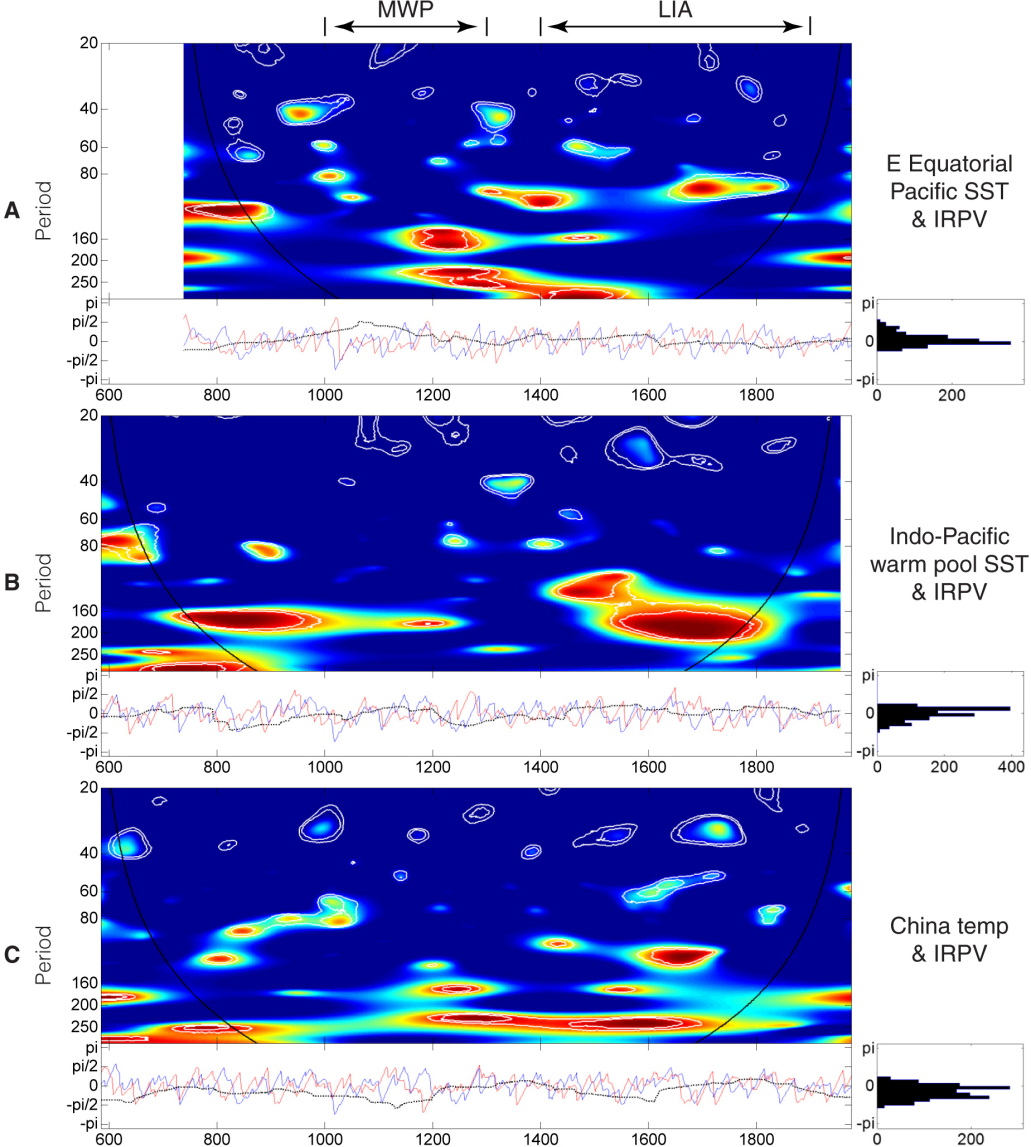
Wavelet coherency between the IRPV index and Equatorial Pacific SST/China-wide land surface temperature. (**A**) Eastern Tropical Pacific Ocean SST [68] and the IRPV. (**B**) Indo-Pacific warm pool SST [69] and the IRPV. (**C**) China-wide land surface temperature [48] and the IRPV. For the upper-left graphs of **A**–**C**, the color code for coherence values varies from dark blue (low values) to dark red (high values). The black curve indicates the cone of influence that delimits the region not influenced by edge effects and the dashed line show the α = 10% significance levels computed based on 1,000 Markov bootstrapped series. For the lower-left graphs of **A**–**C**, the dotted lines represent phase difference; the red line represents the phase of the SST/land surface temperature considered; and the blue lines represent the phase of IRPV. For the lower-right graph of **A**–**C**, the distribution of the phase difference of the two considered time-series is shown.

We calculate the wavelet coherency between the Eastern Tropical Pacific Ocean SST (derived from diatom records in El Junco Lake, Galápagos and spanned AD731–2004) [68] and the IRPV index. Only intermittent 160+ year bands are found (Fig 9A). In parallel, we calculate the wavelet coherency between the Indo-Pacific warm pool SST (derived from sediment cores in Makassar Strait, Indonesia and spanned AD20–1959) [69] and the IRPV index (Fig 9B), and identify a significant ~160–200 year band. The band is relatively stable, except its periodicities weaken in the MWP–LIA transition as well as the transition between the LIA and the warm 20th century. Interestingly, during the above transitions, the 160+ year periodicities in the wavelet coherency between the Eastern Tropical Pacific Ocean SST and IRPV are relatively strong. This suggests that although the Eastern Tropical Pacific Ocean SST itself may not be a very important factor, it supplements the Indo-Pacific warm pool SST in driving the IRPV in NW China. As the periodicities in our wavelet coherency results seem to be modulated by the alternation of climatic episodes, we also compare the IRPV index with China-wide land surface temperature (derived from multi-proxies via Partial Least Square regression method and spanned AD1–1999) [48]. Relatively continuous ~250 year periodicities are found in their coherency (Fig 9C). Briefly, the Indo-Pacific warm pool SST and China-wide land surface temperature is revealed to be the prominent driver of IRPV in NW China at the centennial to multi-centennial time-scale.

We also compute the phases of the two time-series to obtain information about the level of coherency. No significant phase difference is found, as the distribution of phase difference is almost centered at zero. This implies that those statistically-significant periodicities in the above sections are synchronous.

## Discussion

Our main findings are recapitulated as follows for further discussion:

First, the authors composed an index to track the IRPV in NW China, which is robust in tracing the precipitation variation between the arid/semi-arid and humid regions in, as well as revealing the influence of ASM on, NW China.

Second, the IRPV in NW China is associated with anomalous periodicities/unusually strong fluctuations in the LIA, which may be caused by the change of hydro-climatic regime in NW China during the period.

Third, at the multi-decadal to centennial time-scale, the variability of the IRPV in NW China is dominated by ENSO. At the centennial to multi-centennial time-scale, the variability of the IRPV is dominated by the Indo-Pacific warm pool SST and China-wide land surface temperature.

### ENSO and the IRPV

The mechanism that links ENSO and the IRPV in NW China is the influence of ENSO on the intensity and position of the Western Pacific sub-tropical high and consequently on ASM precipitation. Wang and Li [70] note that when sea surface temperature in the Eastern Equatorial Pacific becomes warmer during El Niño years, the meridional temperature gradient will become larger, thus implying a stronger Hadley cell. A stronger Hadley circulation will induce a stronger intensity of the Western Pacific sub-tropical high, which together with a westward shift of the location of the sub-tropical high can lead to a decrease in the extent of the northern latitude of ASM and result in a decrease of the precipitation in the arid region of NW China [71–73]. Also, the westward shift of the Western Pacific sub-tropical high facilitates the respective dominance of the east South Asia High pattern and a plateau vortex/trough in the high and the middle layers of the troposphere over the Tibetan Plateau and its northern neighboring region. This forms a “high upper layer and low lower layer” pressure field, which further suppresses rainfall in the arid region of NW China [27]. Such ENSO-induced regional variation of hydro-climate is imperative in determining the IRPV in NW China.

### Indo-Pacific warm pool SST and the IRPV

Pacific Ocean SST plays a significant role in modulating the hydro-climate in NW China and the precipitation disparity between the Westerlies-dominated and the ASM-dominated regions in East Asia [18, 27]. Also, our IRPV index strongly connects with the Indo-Pacific warm pool SST rather than the Eastern Tropical Pacific Ocean SST. The Indo-Pacific warm pool is the largest and warmest sea surface water body on the Earth [69, 74]. On one hand, as shown in meteorological records, increasing Western Tropical Pacific SST pushes the sub-tropic high over East Asia to the north, resulting in the northward shift of the monsoon fringe and its associated rain-belt [74]. At longer time-scale, lower SST in the Indo-Pacific warm pool is associated with weaker ASM in China [18, 69]. On the other hand, recent studies show that the Indo-Pacific warm pool SST negatively correlates with the precipitation in the Westerlies-dominated region in NW China [75]. The negative correlation may be attributable to the high pressure anomaly over the Indian and Western Pacific Oceans and its associated southwesterly moisture flux [75], but the related mechanism remains to be further investigated. The above synthesis implies that the warming of the Indo-Pacific Ocean can magnify the precipitation disparity between the Westerlies-dominated and the ASM-dominated regions (i.e., IRPV) in NW China. Although the hydro-climatic influence of ENSO and the Indo-Pacific warm pool SST on NW China is usually interwoven [75], it should be highlighted that the lower SST in Indo-Pacific warm pool in the LIA is not caused by El Niño events or an El Niño-like mean Pacific state, but by the propagation of cool North Pacific surface water via the South China Sea or Java Sea pathway [69]. Together with the relatively weak coherency between the Eastern Equatorial Pacific Ocean SST and IRPV (Fig 9A), the Indo-Pacific warm pool SST may be a forcing that is somehow independent of ENSO in driving the IRPV in NW China. On the other hand, the change of monsoon precipitation brought by the Indo-Pacific warm pool SST will have feedback effect on the SST itself, which alters the sea surface latent heat fluxes and consequently ENSO activities [74, 76]. This may explain the presence of the 160+ year band in the ENSO–IRPV coherency (Fig 8E).

### Long-term temperature change and the anomalous periodicities of the IRPV

Long-term temperature change affects the hydro-climate in NW China through the influence on the strength and the relative positions of ASM and Westerlies. In the LIA, the lower temperature over the Tibetan Plateau and the Northern Hemisphere reduce the land-sea thermal contrast between the Asian continent and the North Pacific [4]. The thermal effect of the Tibetan Plateau is especially strong for the East ASM [77]. In addition, lower hemispheric temperature in the LIA also drives the Pacific Inter-tropical Convergence Zone south of its modern position by as much as 500 km [78]. As a result, the lower-troposphere low-pressure system over eastern Asia weakens, and the western Pacific sub-tropical high weakens, with its location shifting southward. Consequently, the ASM is weakened and the Winter Monsoon is strengthened, which causes the north fringe of ASM to shift southward. The decrease of temperature also forces the Westerlies to migrate southward [4, 24, 40].

It should be noted that the hydro-climatic effect of other ocean-atmospheric modes upon NW China is also contingent upon the relative strength of ASM and Westerlies [30, 32, 33, 79]. The southward migration of ASM in the LIA may debilitate the role of AMO on the aridity threshold variability in NW China during the time, as its hydro-climatic effect is mainly operationalized via the change of ASM precipitation [27]. On the other hand, the southward migration of Westerlies in the LIA facilitates the coupled hydro-climatic influence of the Atlantic Ocean and the Arctic Ocean on NW China, in which negative NAO conditions reduced the difference in sea-level pressure between Iceland and the Azores, suppressing the Westerlies. This allows Arctic dry air to propagate southwards into Asia, and *vice versa* [75]. Hence, the hydro-climate in NW China simultaneously interacts with the three ocean-atmosphere systems (i.e., Pacific, Atlantic, and Artic) during the period. This makes the hydro-climatic regime in NW China very unstable, especially in the region north of the fringe of ASM as revealed by the unusually strong ~120–200 year periodicities in the wavelet power spectrum of PI_A_ (Fig 6B). Besides, the above mechanism, which is modulated by the long-term temperature change, may account for the presence of long ~250 year periodicities in the coherency between China-wide land surface temperature and IRPV index (Fig 9C).

### Knowledge contribution

The long-term hydro-climatic influence of various ocean-atmospheric modes upon different localities of NW China has been mentioned in previous paleo-climate/environment studies [6–9, 11, 18, 24, 32, 33, 40]. Yet, their effect in driving the IRPV in NW China has rarely been addressed. By composing the decadal IRPV index and applying wavelet analysis, we present new evidence about the unusual climatic periodicities in NW China during the LIA mentioned in previous studies [21, 58], which is not only characterized by increasing amount of precipitation in the arid/semi-arid regions of NW China throughout the LIA [25, 59], but also characterized by intense variations in precipitation between the arid/semi-arid and the humid regions in NW China within the period. In addition, we statistically substantiate ENSO as the prominent multi-decadal to centennial driving force, while the Indo-Pacific warm pool SST and China-wide land surface temperature is the prominent centennial to multi-centennial driving force, for the IRPV in NW China.

While some meteorological [72, 74, 80–83] and paleo-climate/environment studies [6–9, 11] also highlight the tele-connection between ENSO activities and precipitation anomalies in NW China, the teleconnection is often demonstrated to be inter-annual to decadal (i.e., at a frequency of 2–10 year). In this study, we find that ENSO can drive the regional precipitation disparity in NW China at a frequency of ~60–80 years. Even though some scholars have mentioned the tele-connection between ENSO activities and precipitation change in NW China and the regions nearby at the long-term temporal scales [27, 79, 84], the relationship in the pre-instrumental period has not been validated quantitatively. Here we present the quantitative validation of the low-frequency tele-connection between ENSO and precipitation change in NW China covering both pre-instrumental and instrumental periods. The tele-connection is found to be a consistent feature inherent to the climatic regime in NW China, which can be traced back at least to the early 14^th^ century.

## Conclusions

The topic of long-term IRPV in NW China [4] and the whole of China and its surrounding regions [27] has been addressed in recent paleo-climate/environment studies. We take a step forward by composing a relatively fine-grained index (in decadal resolution) to quantify the IRPV in NW China over the past 1,400 years. Our study area overlaps with the present-day northern fringe of ASM, which is the physical divide between semi-arid/arid areas and humid areas in China. In addition, we quantitatively validate its association with various driving forces.

The regime shifts for the coherency between the IRPV index and ocean-atmospheric modes, together with the significant coherency between the IRPV index and China-wide land surface temperature, reveal that long-term temperature change is an imperative factor in modulating the ocean-atmosphere coupling [30, 32, 33] and consequently the IRPV in NW China at the multi-decadal to multi-centennial time-scales. Average global temperature has been rising since the AD1980s. The warmth is unprecedented over the past two millennia [65, 66], resulting in a more vigorous hydrological cycle [72]. The hydro-climatic regime in NW China is further complicated by the periodic decoupling of the East ASM and Indian Summer Monsoon in their changes [85]. Owing to the interaction between the East ASM and the Westerlies at the northern fringe of ASM, variations in the aridity threshold regime triggered by atmospheric circulation changes will be more pronounced in NW China than in other monsoon regions on the Earth [32]. The spatial heterogeneity of climatic regimes within NW China in recent decades should be thoroughly investigated [86].

In the present study, we apply wavelet analysis to examine the long-term IRPV in NW China in AD580–1979 and examine the major driving forces behind it. Our method is notably free from the assumption of stationarity, helping us to interpret multi-scale, non-stationary time-series data and reveal features we could not see otherwise. This is critically important in examining how gradual change is forced by exogenous variables [42, 43]. We make a pioneering effort to present a fine-grained picture and also examine quantitatively the long-term IRPV in NW China. Admittedly, our results are primarily based on the inhabited regions in NW China. It is necessary for us to further expand the geographic coverage of our historical datasets, or combine them with other proxies [40, 52], to investigate the IRPV across the entire NW China over an extended time span. These efforts may help us to better understand problems such as the differential trends of hydro-climatic changes in the eastern and western parts of NW China since the AD1980s [7, 86].

## Supporting Information

S1 Table Complete PI_A_, PI_B_, and IRPV index. (DOCX)
