# Supplementary material for: Quantifying the Intra-Regional Precipitation Variability in Northwestern China over the Past 1,400 Years
Source: PLoS One. 2015 Jul 8;10(7):e0131693. doi: 10.1371/journal.pone.0131693 (PMC4495927; doi:10.1371/journal.pone.0131693)
Supplement: S1 Table — (DOCX) [file pone.0131693.s001.docx]

**S1 Table.** **Complete PI_A_, PI_B_, and IRPV index.**

| **Year** | **PI_A_** | **PI_B_** | **IRPV** |
| --- | --- | --- | --- |
|  |  |  |  |
| 585 | –2.00 | –2.00 | 0.00 |
| 595 | –2.00 | –2.00 | 0.00 |
| 605 | 0.00 | 0.00 | 0.00 |
| 615 | –2.00 | –0.67 | –1.33 |
| 625 | –2.00 | –0.67 | –1.33 |
| 635 | –1.00 | –1.00 | 0.00 |
| 645 | –0.67 | 0.00 | –0.67 |
| 655 | –1.43 | –0.40 | –1.03 |
| 665 | –2.00 | –1.20 | –0.80 |
| 675 | –0.40 | –0.86 | 0.46 |
| 685 | –2.00 | –1.00 | –1.00 |
| 695 | –2.00 | –2.00 | 0.00 |
| 705 | –2.00 | –0.33 | –1.67 |
| 715 | –0.50 | –0.55 | 0.05 |
| 725 | 0.00 | 1.20 | –1.20 |
| 735 | –0.67 | 0.29 | –0.95 |
| 745 | 2.00 | 0.67 | 1.33 |
| 755 | –1.20 | –0.40 | –0.80 |
| 765 | 0.00 | 0.33 | –0.33 |
| 775 | –1.00 | –0.22 | –0.78 |
| 785 | –2.00 | –0.67 | –1.33 |
| 795 | –1.00 | –1.11 | 0.11 |
| 805 | 0.67 | 0.29 | 0.38 |
| 815 | –2.00 | 0.00 | –2.00 |
| 825 | –0.67 | 0.33 | –1.00 |
| 835 | –2.00 | –0.46 | –1.54 |
| 845 | –1.20 | –0.29 | –0.91 |
| 855 | –0.40 | 0.00 | –0.40 |
| 865 | 0.00 | 0.00 | 0.00 |
| 875 | –2.00 | –2.00 | 0.00 |
| 885 | 0.67 | 0.67 | 0.00 |
| 895 | 0.00 | 0.67 | –0.67 |
| 905 | –0.40 | –0.86 | 0.46 |
| 915 | –2.00 | –2.00 | 0.00 |
| 925 | –2.00 | –1.50 | –0.50 |
| 935 | –1.50 | –1.11 | –0.39 |
| 945 | –2.00 | –0.86 | –1.14 |
| 955 | –2.00 | –2.00 | 0.00 |
| 965 | –2.00 | –1.00 | –1.00 |
| 975 | –2.00 | –0.67 | –1.33 |
| 985 | 0.00 | 2.00 | –2.00 |
| 995 | 0.00 | 0.00 | 0.00 |
| 1005 | –1.20 | 0.67 | –1.87 |
| 1015 | –2.00 | 0.50 | –2.50 |
| 1025 | –2.00 | –0.86 | –1.14 |
| 1035 | –2.00 | –2.00 | 0.00 |
| 1045 | –2.00 | –1.33 | –0.67 |
| 1055 | –2.00 | –1.33 | –0.67 |
| 1065 | –1.00 | –1.20 | 0.20 |
| 1075 | –1.56 | –1.27 | –0.28 |
| 1085 | –2.00 | –2.00 | 0.00 |
| 1095 | –1.33 | –1.33 | 0.00 |
| 1105 | –2.00 | –0.67 | –1.33 |
| 1115 | 0.00 | –2.00 | 2.00 |
| 1125 | –2.00 | –2.00 | 0.00 |
| 1135 | 0.00 | 0.00 | 0.00 |
| 1145 | –2.00 | –2.00 | 0.00 |
| 1155 | 0.00 | 2.00 | –2.00 |
| 1165 | –2.00 | –2.00 | 0.00 |
| 1175 | –0.67 | –0.50 | –0.17 |
| 1185 | –0.67 | –0.29 | –0.38 |
| 1195 | –2.00 | –0.33 | –1.67 |
| 1205 | –1.43 | –0.80 | –0.63 |
| 1215 | –2.00 | –2.00 | 0.00 |
| 1225 | –2.00 | –1.43 | –0.57 |
| 1235 | 0.00 | 2.00 | –2.00 |
| 1245 | 0.00 | 0.00 | 0.00 |
| 1255 | 0.00 | 0.00 | 0.00 |
| 1265 | 0.00 | –2.00 | 2.00 |
| 1275 | 0.00 | –2.00 | 2.00 |
| 1285 | –2.00 | 0.00 | –2.00 |
| 1295 | 0.00 | 0.00 | 0.00 |
| 1305 | –1.00 | –2.00 | 1.00 |
| 1315 | –0.40 | 0.40 | –0.80 |
| 1325 | –0.67 | –0.15 | –0.51 |
| 1335 | –1.00 | 0.22 | –1.22 |
| 1345 | 0.00 | 0.40 | –0.40 |
| 1355 | 0.00 | –2.00 | 2.00 |
| 1365 | –2.00 | –2.00 | 0.00 |
| 1375 | –2.00 | –1.43 | –0.57 |
| 1385 | 0.00 | 0.40 | –0.40 |
| 1395 | 0.00 | 2.00 | –2.00 |
| 1405 | –2.00 | –1.20 | –0.80 |
| 1415 | –2.00 | 0.67 | –2.67 |
| 1425 | –0.67 | –1.43 | 0.76 |
| 1435 | 0.00 | 0.22 | –0.22 |
| 1445 | 0.00 | –0.91 | 0.91 |
| 1455 | –1.11 | –1.20 | 0.09 |
| 1465 | –1.11 | –0.33 | –0.78 |
| 1475 | –1.43 | 0.67 | –2.10 |
| 1485 | –2.00 | –2.00 | 0.00 |
| 1495 | –2.00 | –2.00 | 0.00 |
| 1505 | 0.40 | 2.00 | –1.60 |
| 1515 | –0.67 | 2.00 | –2.67 |
| 1525 | –2.00 | –1.33 | –0.67 |
| 1535 | –1.20 | 0.00 | –1.20 |
| 1545 | 0.40 | 2.00 | –1.60 |
| 1555 | 0.67 | 0.67 | 0.00 |
| 1565 | 0.00 | –2.00 | 2.00 |
| 1575 | 2.00 | 2.00 | 0.00 |
| 1585 | –0.22 | –1.50 | 1.28 |
| 1595 | 2.00 | 0.00 | 2.00 |
| 1605 | 0.86 | 0.00 | 0.86 |
| 1615 | 0.29 | 0.00 | 0.29 |
| 1625 | 0.00 | –0.40 | 0.40 |
| 1635 | –1.43 | –0.67 | –0.76 |
| 1645 | –2.00 | 0.00 | –2.00 |
| 1655 | 0.00 | 0.50 | –0.50 |
| 1665 | –0.40 | 2.00 | –2.40 |
| 1675 | 0.00 | 2.00 | –2.00 |
| 1685 | –2.00 | 1.00 | –3.00 |
| 1695 | 2.00 | –2.00 | 4.00 |
| 1705 | –2.00 | 1.20 | –3.20 |
| 1715 | –2.00 | –2.00 | 0.00 |
| 1725 | –1.11 | –0.67 | –0.44 |
| 1735 | 2.00 | 0.86 | 1.14 |
| 1745 | –0.50 | 0.67 | –1.17 |
| 1755 | 0.67 | 1.00 | –0.33 |
| 1765 | –0.80 | –0.67 | –0.13 |
| 1775 | –0.86 | 0.67 | –1.52 |
| 1785 | 0.40 | 0.67 | –0.27 |
| 1795 | –1.00 | –2.00 | 1.00 |
| 1805 | 1.60 | 0.29 | 1.31 |
| 1815 | 0.00 | 2.00 | –2.00 |
| 1825 | 0.29 | 2.00 | –1.71 |
| 1835 | –2.00 | 0.67 | –2.67 |
| 1845 | –0.86 | 0.40 | –1.26 |
| 1855 | 1.20 | 0.67 | 0.53 |
| 1865 | –0.22 | 0.00 | –0.22 |
| 1875 | –1.33 | –0.29 | –1.05 |
| 1885 | 0.86 | 1.20 | –0.34 |
| 1895 | 0.00 | 0.50 | –0.50 |
| 1905 | –0.40 | –0.29 | –0.11 |
| 1915 | –0.67 | 0.00 | –0.67 |
| 1925 | –1.11 | –1.00 | –0.11 |
| 1935 | –0.13 | 0.80 | –0.93 |
| 1945 | 0.13 | 0.29 | –0.15 |
| 1955 | 0.18 | 0.40 | –0.22 |
| 1965 | –0.18 | 0.00 | –0.18 |
| 1975 | –0.33 | –1.00 | 0.67 |
|  |  |  |  |
